# NodePM: A Remote Monitoring Alert System for Energy Consumption Using Probabilistic Techniques

**DOI:** 10.3390/s140100848

**Published:** 2014-01-06

**Authors:** Geraldo P. R. Filho, Jó Ueyama, Leandro A. Villas, Alex R. Pinto, Vinícius P. Gonçalves, Gustavo Pessin, Richard W. Pazzi, Torsten Braun

**Affiliations:** 1 Institute of Mathematics and Computer Science, University of São Paulo, São Carlos-SP 13566-590, Brazil; E-Mails: joueyama@icmc.usp.br (J.U.); vpg@icmc.usp.br (V.P.G.); 2 Institute of Computing, University of Campinas, Campinas-SP 13083-852, Brazil; E-Mail: leandro@ic.unicamp.br; 3 Department of Computer Science and Statistics, São Paulo State University, São José do Rio Preto-SP 15054-000, Brazil; E-Mail: arpinto@ibilce.unesp.br; 4 Vale Institute of Technology (ITV), Belém-PA 66055-090, Brazil; E-Mail: gustavo.pessin@vale.com; 5 Faculty of Business and Information Technology, University of Ontario Institute of Technology, Oshawa, ON 2000, Canada; E-Mail: richard.pazzi@uoit.ca; 6 Institute of Computer Science and Applied Mathematics, University of Bern, Bern 3012, Switzerland; E-Mail: braun@iam.unibe.ch

**Keywords:** smart grid, wireless sensor networks, electronic equipment, energy consumption, feedback

## Abstract

In this paper, we propose an intelligent method, named the Novelty Detection Power Meter (NodePM), to detect novelties in electronic equipment monitored by a smart grid. Considering the entropy of each device monitored, which is calculated based on a Markov chain model, the proposed method identifies novelties through a machine learning algorithm. To this end, the NodePM is integrated into a platform for the remote monitoring of energy consumption, which consists of a wireless sensors network (WSN). It thus should be stressed that the experiments were conducted in real environments different from many related works, which are evaluated in simulated environments. In this sense, the results show that the NodePM reduces by 13.7% the power consumption of the equipment we monitored. In addition, the NodePM provides better efficiency to detect novelties when compared to an approach from the literature, surpassing it in different scenarios in all evaluations that were carried out.

## 1. Introduction

In the last few years, there has been a growing demand for electricity on the part of industries, commercial establishments and residential dwellings. This has been the situation in both in Brazil and the rest of the world. In the period 2000–2010, the per capita energy consumption in Brazil and in the world increased by approximately 25% and 24%, respectively [1]. Therefore, such a scenario requires a more intelligent electricity system, which can allow energy consumption to be reduced in every piece of electronic equipment and encourage consumers to employ efficient strategies for the reduction of energy consumption.

Information technology for the electric power system, integrated to the systems of communication and electrical network infrastructure, known as the smart grid [2], allows us to monitor and manage the electrical power system, anywhere and anytime. In this sense, the use of smart grids has become increasingly important in the urban scenario, as they offer the integration of various energy sources, such as hydroelectric, atomic, solar and wind power.

It is expected that the use of the smart grid will become a reality in the next few years, since industry, universities and governments throughout the world have earmarked funds for the development of this technology. This trend can be confirmed by the existence of different projects and government initiatives at both national and international levels, as well as in industry and the academic world, which are devoted to smart grids [2–7].

Despite all the achievements in this area, typically, the electric power utilities provide only the total energy consumption spent on a residence and do not offer, for example, support for remotely managing the power consumption of electronic equipment. This means more costs for companies. In addition, it is not a simple task to determine which piece of electronic equipment have the greatest influence on the electricity bill.

Thereby, an alternative to overcome some electricity suppliers' service limitations is to integrate a wireless sensors network (WSN) into the electronic equipment of the residences [3,6,8–12]. With the aid of a WSN, a system can be established aiming at monitoring the use of each household socket in real time, consequently allowing the user to know his track record in terms of energy consumption. Thereafter, the user can find out punctually whether there is any type of waste and take proper actions. Detailed energy consumption information can also be used to provide the household with optimal energy saving times in countries where utility companies offer distinct time-of-use (TOU) rates, for example, being able to take advantage of off-peak pricing.

Studies show that providing information about how, when, where and what users are using can help us to make the right decisions [13–15]. Hence, it is of crucial importance to use intelligent methods in the smart grids for novelty detection and to inform the users in an individual and autonomous way, when some anomaly has occurred in the energy consumption of electronic equipment. These anomalies can arise, for example, when a piece of equipment consumes more energy than expected and/or when the equipment begins to behave in a way outside the normal pattern.

In this context, we propose an intelligent method, named the Novelty Detection Power Meter (NodePM), to detect the novelties in the energy consumption of electronic equipment monitored by a smart grid. Considering the entropy of each device monitored, which is calculated based on a Markov chain model, the proposed method detects the novelties through a machine learning (ML) algorithm. As proof of concept, the NodePM is integrated into a platform for the remote monitoring of energy consumption, which consists of a WSN. Thus, it is possible to intelligently send standalone alerts to users (for example, on a smartphone) when something anomalous happens in the electronic equipment.

In this sense, this work differs from existing solutions in at least three aspects: (i) it employs an intelligent method to monitor electric energy consumption; (ii) it uses ML techniques to analyze the behavior of electronic equipment by means of the WSNs; and (iii) it sends alerts to a smartphone in an intelligent way when an anomaly is identified.

To validate the performance of the NodePM, we built a prototype (see Section 3) to scan the power consumption of the electronic equipment. A number of experiments were conducted with this prototype, including an analysis of variance (ANOVA) and parametric and non-parametric tests. The results of these tests, which were obtained from a statistical analysis, provided evidence of the feasibility of the NodePM in the platform that was developed. In addition, the NodePM provided better efficiency to detect novelties when compared with the Self-Organizing Novelty Detection (SONDE) [16] method, outperforming it in different scenarios in all evaluations carried out.

The rest of this paper is structured in the following way. Section 2 reviews related studies. Section 3 describes the strategy employed for the development of the work and the method proposed. Sections 4 and 5 provide an assessment of the performance and include a discussion of the results obtained. Finally, Section 6 shows the conclusions and examines what contribution has been made by this study.

## 2. Related Work

Several studies have been published in the area of smart grids in the last few years. This section presents scientific papers [3,5,6,8,9,11,12] whose focus corresponds mostly to the monitoring of electric energy consumption. However, despite all the recent advances that have been made, there are still a number of challenges and unresolved problems in this area. For example, the lack of a methodology to detect anomalies/novelties in the monitored environment.

One of the oldest models of a smart grid is the Telegestore project [6]. Telegestore is a system for the remote management of residential and commercial meters with a view toward exploring the low-voltage distribution system between the transformers and meters. The main limitations of Telegestore are as follows: (i) the disregard of a method for detecting anomalies (for example, black-outs) in the low voltage system; and (ii) the monitoring of the sectors is not examined, such as a room in a house, for example.

Erol-Kantarci and Mouftah [5] propose the use of a WSN to manage the electricity of a residence by means of the smart grid. For this reason, researchers have proposed a work, called Appliance Coordination (ACORD), to reduce the cost of energy at peak times. The main limitations of the ACORD are as follows: (i) it does not make use of the benefits that the WSNs offer (for example, monitoring for sectors); (ii) there is a lack of any method for novelty detection in the monitored environment; and (iii), finally, the evaluation of ACORD is carried out through simulations.

In Brazil, there have also been some measures taken with regard to the smart grids, for example, The Center for End-user Monitoring (CMUF) [12]. The aim of the CMUF is to assist people in monitoring electric energy consumption in real time and in incorporating a low-cost system. However, the main limitations that were found in CMUF are as follows: (i) to not to integrate into the platform a method of novelty detection in the monitored environment; and (ii) due to its centralized nature, the system might be vulnerable to data overload.

The work that most closely resembles our proposal is that of [11]. The researchers propose a smart meter for the measurement of electric energy in a residential dwelling. The meter carries out the monitoring of the electricity by showing which pieces of equipment in the dwelling consume the most energy. In spite of its similarities, the smart meter has the following drawbacks: (i) the platform does not use a method for novelty detection in the electric equipment; and (ii) it does not use a cloud server to assist in information management.

## 3. Remote Monitoring System for Energy Consumption in a Residential Dwelling

This section outlines our proposal for the remote monitoring of electric energy consumption in a residential context. A number of stages were necessary to carry out this work, the principal being: (i) the development of a platform that employs a WSN as proof of concept; (ii) the implementation of the NodePM in the platform developed to detect novelties in the environment being monitored; and (iii), finally, the development of a front-end application to allow the consumer to visualize the information about the energy consumption of each electronic equipment in the dwelling.

Thus, it should be stressed that the platform that is developed and examined in the following section is simple to install and easy to use, which means there is no need to have a specialist in the area. The NodePM is integrated into the platform with the aim of detecting novelties in the monitored environment. In addition, the developed system allows for the monitoring of the electric energy consumption anywhere and anytime.

### 3.1. Platform Constructed Using WSN

The proposed platform consists of three stages:

- Stage 1 is responsible for energy consumption data acquisition with regard to the electronic equipment, by means of a WSN.
- Stage 2 is concerned with the data collection procedures in Stage 1. For that reason, a gateway and cloud application (*app engine* [17]) was designed and developed.
- Finally, Stage 3 is responsible for making information available about electric energy consumption for interested parties. To this end, an application for a smartphone was developed.

**Figure 1.**
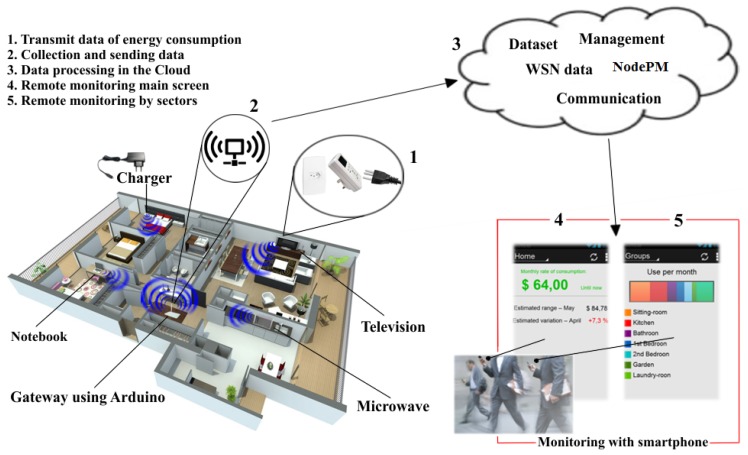
Operational scenario of the platform. WSN, wireless sensors network; NodePM, Novelty Detection Power Meter method.

Figure 1 shows the functions of the remote monitoring platform for electric energy consumption. To this end, we built WSN prototypes from the assembly of wattmeters (the equipment that measures electric energy consumption), Kill-a-Watt, from the company P3 [18]. Wattmeters are directly linked to the wall outlet, and the device that is required for monitoring is connected to it (Label 1, Figure 1). As the wattmeters do not have access to any means of communication, an XBee [19] was added to them, with the aim of allowing the transmission of information about energy consumption. In this way, an Arduino [20] gateway infrastructure was constructed (Label 2, Figure 1) to send information by means of the wireless network technology present in the WSNs and to transmit the data read by the cloud server (Label 3, Figure 1), where the NodePM is deployed (as described in Section 3.2). The server receives the WSN data, processes said data (detecting novelties) and manages (consumption data) the transmission of information, it being possible to do remote monitoring via a smartphone (Labels 4 and 5, Figure 1).

As a result, the user receives the alerts and can visualize the most important information about energy consumption, as presented in Label 4 of Figure 1. The monitoring of the sectors (Label 5, Figure 1) is carried out with regard to the equipment and the room in the house, and/or any area being used, and is only possible on account of the WSN.

In the following subsections, there is an outline of the developed wattmeter and the constructed gateway.

#### 3.1.1. Wattmeter Developed for the Formation of WSN

**Figure 2.**
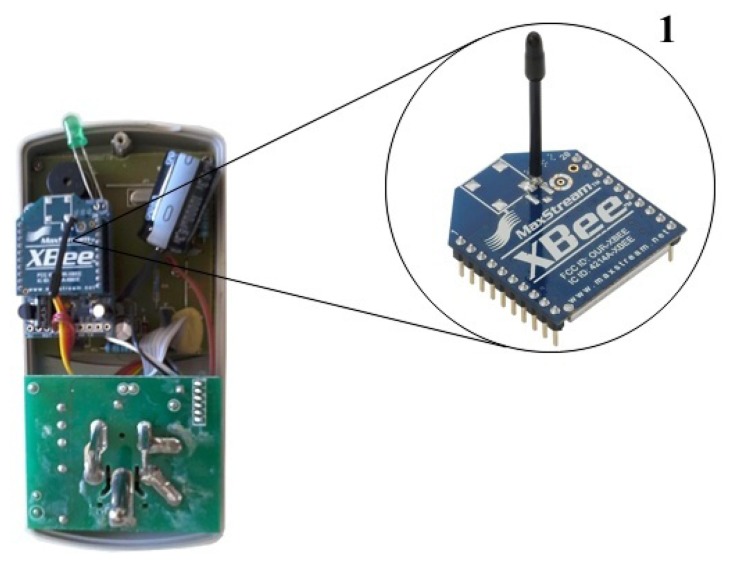
Wattmeter developed with XBee wireless communication for the formation of the WSN.

As one of the stages of this study involves gathering data about the energy consumption of electronic equipments, a wattmeter was developed with wireless communication power, as illustrated in Figure 2, which considers the functions of the WSNs. XBee (Label 1, Figure 2) was integrated into the wattmeter, as a communication module that uses the ZigBee [21] protocol (which is widely used in WSNs). Hence, the developed wattmeter allows the deployment of the WSN, which collects information about the energy consumption of electronic devices.

The choice of the communication module used in the wattmeter (Model XB24-AWI-001) can be explained by the following reasons: (i) low energy consumption; and (ii) an average reach area of 40 m indoors and/or 120 m outdoors.

By means of the employment of the integrated wattmeter circuit, it is possible to know how much a particular piece of equipment is consuming. To this end, through Ohm's Law, it was possible to determine the relationship between the voltage, *V*, and current, *I*, to know the power *P* = *V* × *I*. Therefore, by multiplying the power, *P*, by the time the equipment is in use, we can obtain the energy it consumes in watt-hours.

Thus, it should be underlined that in order to obtain accurate power readings of the equipment in making a transmission, it was established that the XBee would obtain 17 samples of voltage and electric current with 1-ms spacing or, in other words, a sample window of 17 ms. As the sign on the electric line is periodic, *i.e.*, it repeats according to the frequency of the network, which runs at 60 Hz, a wave period will have 1/60 ms, *i.e.*, 16.6 ms of samples. Therefore, our samples sent from 17 ms had enough information for precise readings of the power of the equipment. Moreover, the XBee can be configured to have an identification number, like the MAC address. This identification was used to recognise, for example, which transmitter is connected to the refrigerator or television.

#### 3.1.2. Construction of a Gateway with the Arduino Platform

**Figure 3.**
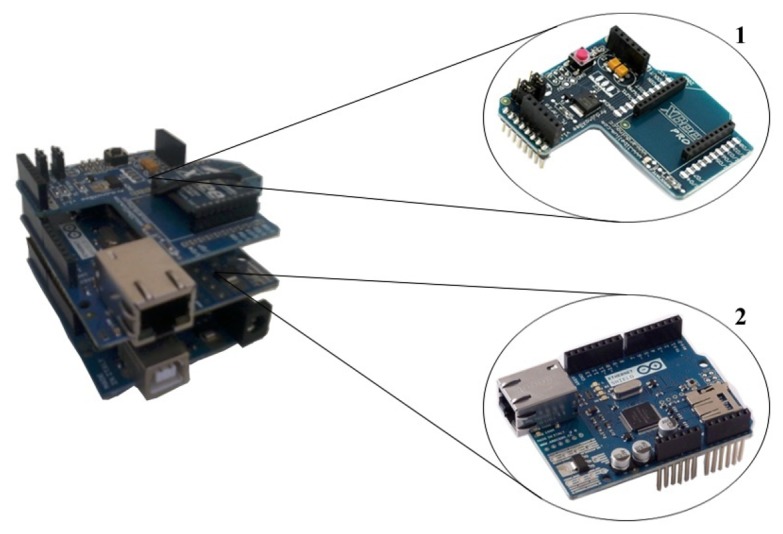
Gateway developed with the Arduino platform using the XBee shield (Label 1) and the Ethernet shield (Label 2).

It was necessary to develop a gateway to gather data from the WSN and transmit it to a cloud server, as illustrated in Figure 3. The Arduino platform was chosen for the development of this gateway, because it is programmable, extensible, portable and has a low consumption of energy.

As the designed gateway needs to communicate with several XBee modules and also have access to an Ethernet network, we needed to enhance the typical Arduino by using shields. Therefore, two shields were employed (one XBee and one Ethernet shield) to allow the connection between the WSN and the cloud server. Thereafter, the XBee shield (Label 1, Figure 3) receives the gathered data from the WSN and transmits it by means of the Ethernet shield (Label 2, Figure 3) to a cloud server.

#### 3.1.3. Implementing Remote Monitoring

We developed an application for an Android-based smartphone to provide users with access to information about energy consumption, as well as to allow them to send warnings whenever a novelty is identified. This is illustrated by Labels 4 and 5 in Figure 1. We adopted an Android-based smartphone for the following reasons: (i) it is open-source software; (ii) it is reasonably lightweight; and (iii), finally, it is widely deployed in smartphones, tablets and laptops.

We adopted Cloud Endpoints to implement the communication between the application and the cloud server. Cloud Endpoint assists in developing an application for Android with a backend targeted to the app engine. In our scenario, the application checks the recent data of energy consumption in the memory device. If it finds no data, the application in the smartphone sets up a communication with the cloud application to retrieve the information requested by the consumers. Moreover, the cloud application processes the data received from the WSN, so that it can detect novelties in the energy consumption of electronic appliances. This step is described in the next section.

### 3.2. Novelty Detection Power Meter (NodePM)

The NodePM is designed by using machine learning concepts, Markov chains and entropy. In Algorithm 1, there is a description of the functions of the NodePM, which consists of three stages:

- The first stage is to process the data with the aid of a classifier, K-nearest neighbors (KNN) (Line 2 of Algorithm 1), aiming to create records of the behavioral state of the electronic equipment.
- The second stage is to capture the element recorded in the first stage and add it to the behavioral state of the Markov chain (Line 3 of Algorithm 1).
- The third stage is to obtain the probability matrix for the Markov chain and calculate the degree of uncertainty of the electronic equipments using entropy variation (Lines 4 and 5 of Algorithm 1).

**Algorithm 1:**
*Novelty Detection Power Meter*.
**Input :** X = (X1,…,Xn);/* dataset */**Output :** alerts to mobile devices**Data:** Threshold, Markov;1**begin**2 State⇐KNN(X);/* classifies unknown instances */3 Markov ⇐ UpdateMarkovChain(Markov, State);/* embeds classified instances in the Markov  chain state */4 Probability⇐GetProbabilidade(Markov);/* probability vector */5 DeltaEntropy⇐Shannon(Probability);/* entropy variation *//* novelty detection in electronic equipment */6 **if** DeltaEntropy ≥ Threshold **then**7  Send(Device);/* send the message to the mobile device */8 **end**9**end**


The stages mentioned above are described in detail in the following subsections to provide a better understanding of both the proposed method and related concepts.

#### 3.2.1. Classification of Behavioral Patterns

This subsection shows the technique that was chosen (KNN) to classify the data gathered by the WSN with the aim of including them in the Markov chain (Section 3.2.2.).

The choice of KNN can be explained by two reasons: (i) in spite of being simple, it has proved to be one of the most effective techniques proposed in the literature; and (ii) it is a traditional and effective technique that can be applied to various classification problems [22].

**Figure 4.**
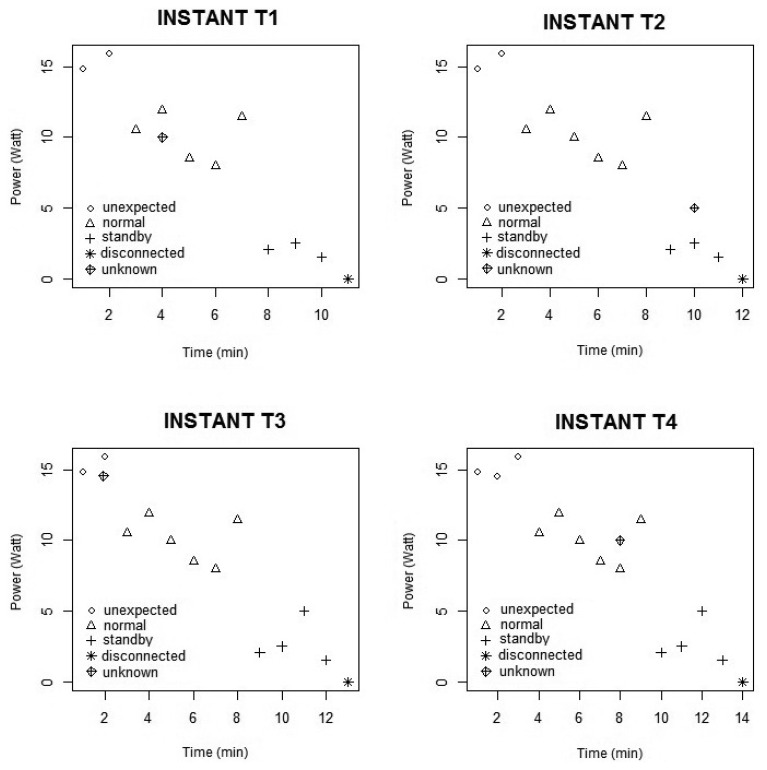
Example of the K-nearest neighbors (KNN) classification.

To facilitate the understanding of the technique and its operation on the platform, Figure 4 illustrates how the gathered data are classified. The graphs in Figure 4 represent the power of a piece of equipment in terms of time (minutes) and allows us to distinguish between four separate classes of energy consumption (standby, disconnected, normal (representing the equipment within a pattern; a refrigerator, for example, is switched on the whole time) and unexpected (representing the equipments outside the pattern and/or consuming energy above the expected level.)).

The KNN classifies unknown instances by analyzing those K-nearest neighbors. In this case, the “unknown” instance in T1 INSTANT(see Figure 4) is classified as a “normal” instance. After the classification, it is of crucial importance to point out that the classified element is added one by one to the training function, with the aim that the method adapts itself in an autonomous way, being capable to operate in a dynamic environment. In this study, adapting to the environment takes place every 5 s, the time stipulated by the platform for sending data. In this way, the T2, T3 and T4 instants follow the same process as T1.

The next stage of the method involves adding the classified instance to each instant of time in a Markov chain state.

#### 3.2.2. Obtaining Behavioral Patterns

**Figure 5.**
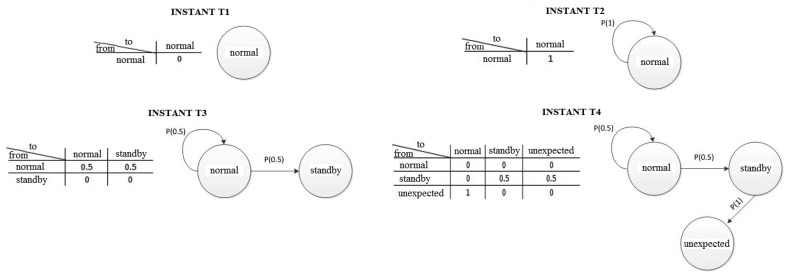
Probability matrix and Markov chain at each instant of time in accordance with Figure 4.

Obtaining behavioral patterns in our model is carried out through the Markov chain [23]. Hence, as the KNN classifies the unknown instances as described in the last stage, a new Markov chain is generated. In Figure 5, the probability matrix and the Markov chain in each of the instants of time are displayed (as shown in Figure 4) with a view toward representing the behavior of the equipment in that instant of time.

For this reason, the probability matrix and the Markov chain are updated to comply with each new classification of KNN (see the parallel between Figures 4 and 5). In this case, in the T1 instant (Figure 5), the first instance is classified as “normal”, although there is no transition in the Markov chain. In the T2 instant (Figure 5), the second instant was also classified as “normal”. However, there is a transition between the first and second classified instant, and as a result, the probability matrix is brought up-to-date and shows a transition **from** the “normal” state **to** the “normal” state. Thus, the Markov chain states are created dynamically in so far as the KNN classifies the behavior of the equipment in that moment of time.

After completing the classification, representing the behavior of the equipment through the Markov chain and obtaining the probabilities for each moment of time, it is necessary to calculate the degree of uncertainty of the equipment by means of Markov chain entropy.

#### 3.2.3. Novelty Detection with the Variation of the Markov Chain Entropy

With the set of probabilities produced by the Markov chain, it becomes possible to calculate the entropy variation of each moment of time by means of the following formula:

$$H(X)=-\sum_{i=1}p(x_{i})\log_{b}p(x_{i})$$

where *log_b_* denotes the logarithm of *x* at base 2 and *p*(*x_i_*) is the probability of an event moving to another state (probability matrix of Figure 5). In this way, each interaction by the user with the equipment produces an energy curve that represents the behavioral alterations of the equipment. In Section 4.1.2., the types of novelty are represented, which have already been found through the proposed algorithm.

## 4. Performance Evaluation

In this section, we present the methodology employed to perform the evaluations. Then, a validation of the proposed method is performed. This validation was divided into two stages. In the first, Section 4.2, a perfomance evaluation is conducted of the NodePM with the Self-Organizing Novelty Detection (SONDE) method. In the second, Section 4.3, the efficiency of the NodePM in detecting anomalies in the energy consumption of electronic equipment is validated.

### 4.1. Methodology

According to [24], each computer system has its own particular features. Thus, the performance evaluation becomes something peculiar to each system being evaluated. For this reason, it is uncommon to use the same evaluation methodology for different computer systems. In the case of this research study, the evaluation carried out comprised four stages, which are as follows: (i) the choice of the baseline for the method; (ii) the types of anomalies encountered; (iii) the definition of the database; and (iv) the monitored environment. Each of these stages is described in the following sections.

#### 4.1.1. The Baseline for the Proposed Algorithm

SONDE [16] is a neural network that adapts its framework of knowledge incrementally (neuron) with the aim of detecting novelties in dynamic environments. SONDE achieves this by classifying similar patterns of entry in the same neuron. If no neuron is able to classify a pattern of entry, a new neuron is created and thus shows a novelty in the environment.

There are four reasons for the choice of SONDE: (i) SONDE can be used in any system and/or application regardless of the database; (ii) the method detects unexpected events in dynamic environments; (iii) the training and the adaptation are undertaken without the intervention of a specialist; and (iv), finally, SONDE achieves a better performance than the Grow When Required [16,25] method.

#### 4.1.2. Types of Anomalies

As stated at the outset, one of the objectives of this study is to detect anomalies in electronic equipment by identifying, for example, electronic equipment that consumes more energy than expected, which can suggest a fault. As the behavioral patterns of each piece of electronic equipment are different, it is not a slight task to discover the anomalies. For example, the energy consumption of a refrigerator is different from that of a television, because of the fact that the former is switched on the whole time, while the latter is only used sporadically. Despite this, by using the NodePM in this research study, two types of novelty/anomaly (qualitative and quantitative) were found, as shown in [26,27]:

- The first occurs when there is an abrupt change in the behavioral pattern of the electronic equipment, *i.e.*, when there is a non-regular state transition, shifting from state *x* to state *y*, caused by a non-expected situation (qualitative).
- The second takes place when the electronic equipment begins to consume more energy than expected during a determined period, *i.e.*, when it changes its usual state from the standpoint of the user (quantitative).

#### 4.1.3. Database

The proposed method employs a dataset containing real information about the interaction of the user with the electronic equipment. This database has the following attributes: (i) the identifier of the equipment in question by the sensor; (ii) the power in watts; (iii) the date of use of the equipment; and (iv) the time the equipment had been used at that moment.

It should be emphasized that the only normalized attribute for the integer type is the date, which represents the number of days elapsed from a fixed date. This attribute is standardized by the platform itself. This uses JavaScript Object Notation (JSON) [28] to transmit the data in a standardized format. Thus, the method does not need to do any normalization of the attributes received.

After sending the data, the samples of the collected dataset were divided into nine partitions. Each partition had a dataset of the days of the weeks. In the first validation, which was carried out in Section 4.2, each element of the set was divided into 25% of the training instances and 75% of the test instances. This technique is known as a hold-out, which the database divides into two sets (a training and test set) [29]. As the objective of the second validation, Section 4.3, is to determine how much the user could reduce energy consumption, there is no need to use the hold-out technique. This way, only the partitions containing the dataset of the days of the weeks were used. In addition, the partitions of the datasets designed for producing results were randomly replicated to ensure there was no degree of dependence in the experiments.

#### 4.1.4. Description of the Monitored Environment

A real environment was created to monitor the energy consumption of electronic equipment in a residential dwelling to produce very accurate results. In this scenario, the equipment was located in different rooms, and for the sake of convenience, the Arduino gateway was beside the router, so that it could communicate with the Internet. The monitoring of the energy consumption was carried out during 2012 (July, August, September, November and December) and 2013 (January, February, June and July). The electronic equipment monitored through the platform was as follows: (i) refrigerator; (ii) router; (iii) coffeemaker; (iv) television; (v) microwave; (vi) shower; (vii) washing machine; and (viii) computer.

### 4.2. Performance Evaluation of the NodePM with the SONDE Method

**Figure 6.**
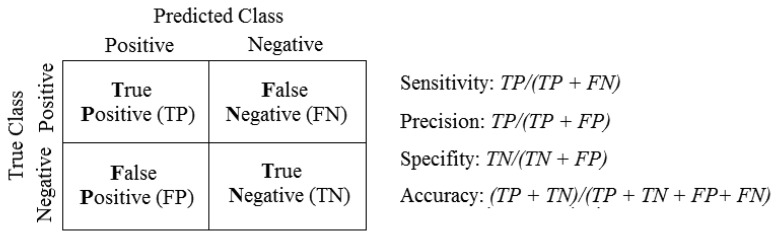
Performance measurements calculated on the basis of the confusion matrix.

This subsection assesses the performance of the NodePM and takes account of the following performance measurements: sensitivity (known by some authors as the detection rate), precision, specificity and accuracy. These measurements, which are shown below, are calculated on the basis of a confusion matrix illustrated in Figure 6, in which the results are evaluated on the basis of the caused losses.

According to [30], these measurements have inherent features, sensitivity being the total of samples whose resulting class is really positive (true positives); precision is the total number of examples classified as positive, but that are not always so or, in other words, true negative; specificity is the opposite of sensitivity and is where it is only necessary to classify as negative the examples that in fact are negative (false positive); and accuracy makes it possible to analyse how precisely the method is able to classify the equipment in a suitable way.

**Figure 7.**
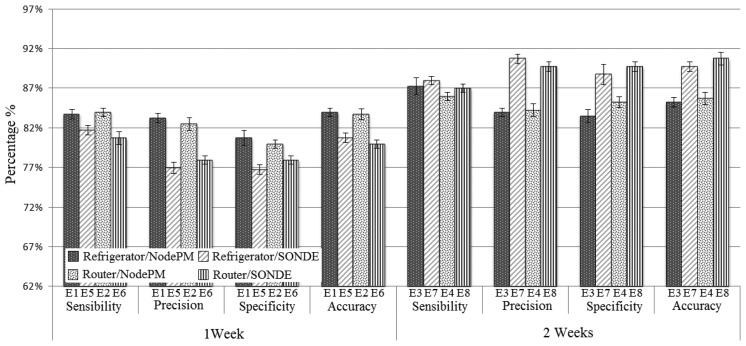
Analysis of the performance of the NodePM on the basis of Table 1.

The sets of parameters established to carry out the performance evaluation are shown in Table 1. The system is evaluated by altering the dataset (one week and two weeks), with the change of the equipment (refrigerator and router) and with an exchange of methods (NodePM and SONDE). These methods employ the dataset that contains real information about the interaction of the user with the equipment. Each set of parameters was executed 11 times with the aim of achieving stability in the results. The results are shown in Figure 7 with a confidence interval of 95% in accordance with the *t*-student distribution.

Figure 7 shows the percentage of the results obtained from the experiments E1 to E8 (Table 1), with regard to the sensitivity, precision, specificity and accuracy measurements for the dataset of one and two weeks. The results highlight the fact that the NodePM has a superior performance with regard to the SONDE when the dataset for one week is considered, regardless of what equipment is used (experiments {(E1,E5),(E2,E6)}). The reason for this was that the NodePM adopted a supervised approach, or in other words, the database had examples that are labelled with a predicted class. Therefore, the method did not need many training instances to achieve good results, because with the dataset of two weeks, its performance was significant (an approximate increase of 3% in the E1 and E4 experiments). However, when the dataset of two weeks is taken into account, the roles are reversed, or in other words, the SONDE has a better performance than the NodePM, regardless of the equipment used (experiments {(E3,E7),(E4,E8)}). This makes sense, for the SONDE is an artificial neural network that is non-supervised and self-organizing. Thus, it is necessary to have a larger dataset, so that the SONDE neurons can be adapted in an incremental way in accordance with the new patterns of entry. For this reason, the improved performance (an increase of ≈ 13% in the E5 and E8 experiments) can be attributed to an increase in the training instances.

#### 4.2.1. Analysis of the Influences of the Parameters

This subsection shows the analysis of the influences of a set of parameters, which are considered in Table 1 with regard to the performance measurements. The analysis was conducted by using a multivariate regression model [24], which includes a causal relationship with more than two variables. In this case, the behavior of a performance measurement can be explained by more than one parameter. Hence, it is possible to analyse which parameters and/or combinations between them has a greater influence on the results.

In Table 2, we present the parameters: methods, dataset and equipment are shown with the respective letters, A, B, and C.

The combinations of two or more letters are the percentages of the interactions between the parameters. It should be noted that the parameter that has most influence over all the performance measurements is the dataset (B), which can also be observed in the results obtained from the pairs of experiments: {(E1,E3), (E2,E4), (E5,E7), (E6, E8)}. The parameter with the second most influence is the combination of the dataset with the method (AB). Such influence makes sense, as it is related to the strong interaction that parameter A has with B. It should be stressed that the parameter with the least influence is the equipment regardless of the set of parameters considered. These statistical assertions provide evidence of the effectiveness of the NodePM in the developed platform.

### 4.3. Performance Evaluation with the NodePM

In this subsection, there is an evaluation of the performance of the NodePM in detecting anomalies in the energy consumption of electronic equipment in a real environment. The objective is to compare the efficiency of the proposed system with that of traditional systems (which, for example, do not provide information about the energy consumption of users).

**Figure 8.**
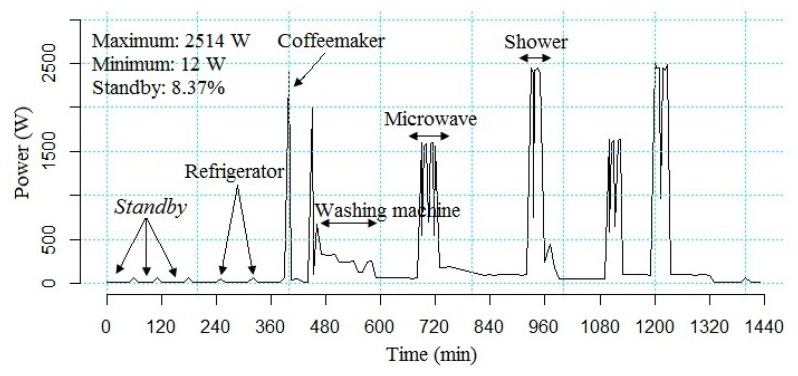
Using the platform developed to observe the energy consumption in terms of waste by the user over a period of 24 h and identifying the idle equipment, those which, for example, remain for a considerable time in standby mode.

The experiments were carried out considering two primary parameters: (i) whether or not our proposal was adopted; and (ii) the period of the monitoring (one, two and three weeks). Since the purpose of the planning is to determine how much the user will be able to reduce his energy consumption, the sum of the energy consumption of the electronic equipment measured in kWh was employed as a response variable. As a result, it was necessary to set out two secondary parameters: (i) types of anomalies (qualitative and quantitative, as described in Section 4.1.2.); and (ii) information about the consumption of energy, as illustrated in Figure 8 (seeking, for example, to identify equipment in the standby mode (electronic equipment in standby mode wastes, on average, 8.37% of energy consumption, according to data collected from the developed platform)). Thus, the configurations of each set of parameters, as summarized in Table 3, were executed 12 times, with experiments A4 till A6 being used as points of comparison.

**Figure 9.**
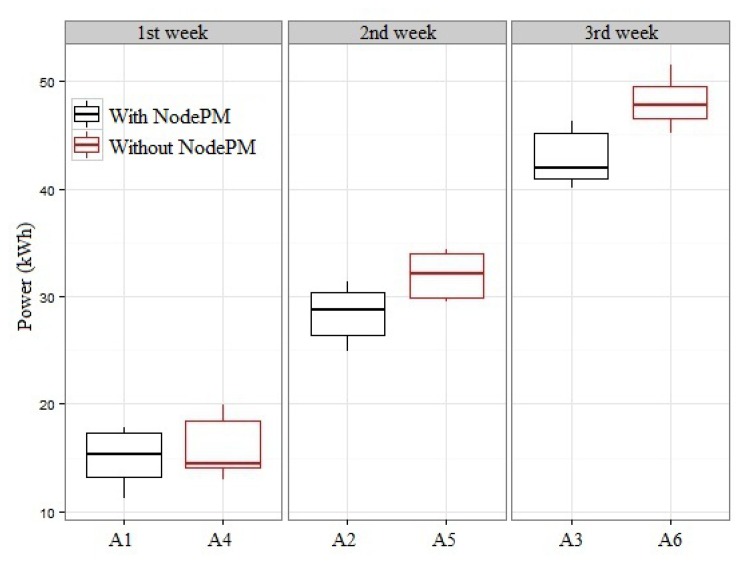
Analysis of the performance of NodePM in accordance with Table 3.

Figure 9 shows the results of energy consumption obtained from experiments A1 to A6 during the monitoring period. Two stages were needed with the purpose of carrying out a statistical validation of the results: (i) a test of the normal adequacy of the sets, aiming to verify if the sets can be considered as normal distributions; and (ii) statistical comparison test between the sets. For the first one, we used the Shapiro–Wilk normality test, which presented the following *p*-values for the sets of A1 to A6: {0.023, 0.300, 0.043, 0.024, 0.009 and 0.790}. Thus, it was noted that with the exception of A2 and A6, the other had the hypothesis of adapting to normality rejected, with a confidence level of 95% being considered. That implies the use of non-parametric methods of comparison between the sets, and for this case, the Wilcoxon rank sum test is indicated (the second stage in the process of statistical validation).

As a result, using the Wilcoxon rank sum test as a test of comparison with the sets, (A1, A4), (A2, A5) and (A3, A6), the following *p*-values were obtained: {0.214, 0.003 e 0.000}, with 95% confidence. Through these results, it was noted that for a set of values for a week, there were no significant statistical differences (experiments A1 and A4 in Figure 9). This situation is derived from the rate of true positives obtained from the experiments {(E1, E5); (E3, E7)} of Table 1. That is, the NodePM was adjusting the model (probability matrix and Markov chain; see Section 3.2) by means of data sent from the WSN. However, statistically, for a period of two and three weeks, there is a reduction of 11.4% and 13.7% of energy consumption (experiments {(A2, A5); (A3, A6)} in Figure 9) when the proposed system is used and, hence, an increase in the rate of true positives of the NodePM ({(E2, E6); (E4, E8)} in Table 1). This shows that the NodePM adjusts the model in an incremental way (dynamic training) through the information sent from the wattmeters and has a satisfactory performance regardless of the period being monitored (winter or summer, for example, Section 4.1.4.) or the equipment being used.

## 5. Discussion of the Results

On the basis of the results obtained from the planning of the experiment in Sections 4.2 and 4.3, some satisfactory considerations can be highlighted.

In the first planning stage (Section 4.2), it was clearly established that the NodePM was feasible in the platform. However, each method has its own peculiar features. In view of this, the choice of the method was an intrinsic aspect of the dataset (see Table 2), since the data collected from the interaction of the user with the equipment in real time, has distinct features. In this way, if the user had equipment that was connected the whole time, such as the refrigerator, router and/or freezer, where there were more data with the interaction, it was most suitable to use SONDE (see Figure 7, experiments E7 and E8). However, if the user interacted with the equipment, such as the microwave oven, the washing-machine and/or coffee-maker, where the equipment is not connected the whole time, there were few interaction data and the most suitable choice is the NodePM (see Figure 7, Experiments E1 and E2).

In the second planning stage (Section 4.3), it was observed that the behavioral changes of the electronic equipment in the long term did not affect the performance of the proposal (see Figure 9, Experiments A2 and A3). For example, the refrigerator tends to show fluctuations in its energy consumption depending on both the time of year and its interaction with the user. This situation was observed during the different periods in which the electronic equipment was monitored (see Section 4.1.4.). In addition, during the experiments, it was noted that the behavior of the users was different at the end of the week compared with weekdays, although this situation did not affect the performance of our proposal.

## 6. Conclusions

In conducting this research study, it was clear that it was important to use a WSN to collect information about electric energy consumption in a residential dwelling. In this scenario, it was observed that there is a need to use novelty detection techniques in determining the energy consumption of electronic equipment. In this sense, we proposed the NodePM, an algorithm that considers the Markov chain entropy with the aid of the KNN to detect novelties. A remote monitoring platform of energy consumption for electronic equipment was developed with the aim of proving the viability of the NodePM.

An extensive assessment and range of experiments in a residential dwelling made it possible to evaluate the efficiency of the NodePM by taking account of different scenarios and parameters. In addition, the results obtained were found to be promising, and two of them were particularly striking: (i) a superior performance with regard to SONDE when the dataset was considered for one week; and (ii) a reduction of energy consumption by 13.7%.

This article has made the following contributions: (i) the use of a WSN for constructing a prototype that monitors the consumption of the energy of electronic equipment individually and in real time; (ii) an intelligent method (NodePM) based on the concepts of ML for novelty detection in a monitored environment; (iii) a front-end system for a smartphone, which receives information about energy consumption by the cloud server and shows the information to the user; (iv) the use of a cloud server as a solution for producing both the information received from the WSNs and regarding the use of the method; and (v), finally, a performance evaluation of the proposed method showing its performance in the detection of novelties in the electronic equipment being monitored by a smart grid. It should be stressed that the implementations and evaluations of this study employ emerging technologies (for example, cloud computing, WSN and smartphones) within the context of a smart grid.

In future studies, we intend to develop a hybrid solution, which can change the methods of novelty detection during the time of execution, depending on the electronic equipment that is employed. In this system, the equipments that are only connected sporadically will use the NodePM, whereas the equipments that are connected the whole time use SONDE.

## Figures and Tables

**Table 1.** Set of parameters chosen to be evaluated. NodePM—Novelty Detection Power Meter method; SONDE—Self-Organizing Novelty Detection method.

**Experiment**	**Method**	**Dataset**	**Equipment**
**E1**	NodePM	1 Week	Refrigerator
**E2**	NodePM	1 Week	Router
**E3**	NodePM	2 Weeks	Refrigerator
**E4**	NodePM	2 Weeks	Router
**E5**	SONDE	1 Week	Refrigerator
**E6**	SONDE	1 Week	Router
**E7**	SONDE	2 Weeks	Refrigerator
**E8**	SONDE	2 Weeks	Router

**Table 2.** Influence of the parameters on the basis of the performance measurements. A, method; B, dataset; C, equipment.

**Performance Measurements**	**A (%)**	**B (%)**	**C (%)**	**AB (%)**	**BC (%)**	**AC (%)**	**ABC (%)**
**Sensitivity**	3.06	81.05	2.25	12.25	0.57	0.25	0.57
**Precision**	0.17	59.14	0.02	39.9	0.07	0.02	0.68
**Specificity**	1.09	77.85	0.82	19.16	0.38	0.12	0.58
**Accuracy**	0.64	64.97	0.07	33.16	0.95	0.01	0.02

**Table 3.** Set of parameters selected for evaluation.

**Experiment**	**Using the Proposed**	**Monitoring Period**
**A1**	Yes	1st Week
**A2**	Yes	2nd Week
**A3**	Yes	3rd Week
**A4**	No	1st Week
**A5**	No	2nd Week
**A6**	No	3rd Week
